# Simultaneous Determination of Purines and Uric Acid in Chinese Chicken Broth Using TFA/FA Hydrolysis Coupled with HPLC-VWD

**DOI:** 10.3390/foods10112814

**Published:** 2021-11-16

**Authors:** Manli Wu, Wangang Zhang, Xixi Shen, Wei Wang

**Affiliations:** 1 College of Food Science and Technology, Nanjing Agricultural University, Nanjing 210095, China; 2020108046@stu.njau.edu.cn (M.W.); wangang.zhang@njau.edu.cn (W.Z.); T2020093@njau.edu.cn (X.S.); 2 National Center of Meat Quality and Safety Control, Nanjing Agricultural University, Nanjing 210095, China; 3 Key Laboratory of Animal Products Processing, Ministry of Agriculture and Rural Affairs, Nanjing 210095, China; 4 Jiangsu Collaborative Innovation Center of Meat Production and Processing, Quality and Safety Control, Nanjing Agricultural University, Nanjing 210095, China

**Keywords:** Chinese chicken broth, purine, uric acid, HPLC, acid hydrolysis, hyperuricemia, gout

## Abstract

Chinese chicken broth is well known for its outstanding nutritional value and flavor, widely consumed in China. This study was designed to develop a sensitive and accurate high-performance liquid chromatography-variable wavelength detector (HPLC-VWD) method to simultaneously determine purines and uric acid in Chinese chicken broth for gout and hyperuricemia dietary management. Chromatographic separation was performed on an Agilent TC-C18 (2) column (4.6 mm × 250 mm, 5.0 µm), using 0.02 M KH_2_PO_4_ (pH 4.0) as a mobile phase. Sample pretreatment was optimized to enable the extraction of all analytes from Chinese chicken broth. The optimal pretreatment conditions were chicken broth-60% trifluoroacetic acid (TFA)/20% formic acid (FA) (1:1, *v/v*) in a volume ratio of 1:3 and hydrolysis for 40 min at 85 °C in a water bath. The limits of detection (LODs) and limits of quantification (LOQs) of the purines and uric acid were 0.58–1.71 µg/L and 1.92–5.70 µg/L, respectively. The recoveries were 91–101%, with the relative standard deviations (RSDs) lower than 3%. The complete method has been successfully applied to determine purines and uric acid in various Chinese chicken soups obtained from different provinces in China.

## 1. Introduction

**Figure 1:**
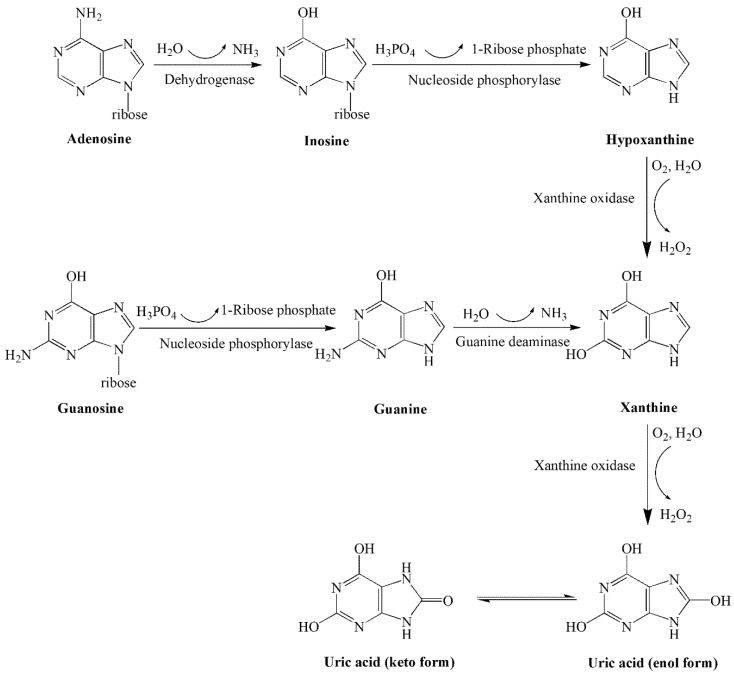
Exogenous purines metabolic pathways.

Purines are natural alkaloids, mainly containing adenine, guanine, hypoxanthine, and xanthine, and widely exist in food in the form of bound purines (nucleic acids, nucleotides, nucleosides) and free bases [1]. When ingested by the body, the end product uric acid is produced by the catalytic action of various enzymes [2] (Figure 1). Once purine metabolism is disturbed, there is an imbalance between uric acid production and renal excretion. In that case, the persistent accumulation of serum uric acid can be deposited as monosodium urate crystals in joints and soft tissues, causing hyperuricemia and even gout [3,4]. Moreover, hyperuricemia can lead to a range of complications such as hypertension, chronic kidney disease, and cardiovascular disease [5,6]. In recent years, the prevalence and incidence of gout have been increasing worldwide, with the highest prevalence in Oceania and reaching over 10% in some specific ethnic groups [7]. According to the 2021 China High Uric Acid and Gout Trend White Paper, the overall prevalence of hyperuricemia in China is 13.3%, with approximately 177 million people being affected and more than 14.66 million suffering from gout [8]. In addition to genetic factors, gout is closely related to people’s dietary and lifestyle habits.

Dietary purines have an essential impact on serum uric acid concentration. As the standard of living improves, foods such as seafood, meat, animal offal, and broth are gradually becoming mainstays of the modern diet. These are high purine foods, and their excessive consumption can disrupt the body’s uric acid balance and increase the risk of hyperuricemia [9]. Furthermore, studies have shown that meat products contain metabolite uric acid in addition to purines, which can directly affect the body’s blood uric acid levels when consumed [10,11]. Chinese chicken broth is a delicious traditional meat broth popular among consumers for its appetite, digestive properties, cold relief properties, and nutritional benefits [12,13,14]. However, besides protein, fat, minerals, and other nutrients, most purines and uric acid are also transferred from the chicken to the soup during the long boiling process. Chicken soup’s flavor and nutritional value often mislead consumers to ignore its uric acid and high purine content. Therefore, the accurate determination of the purine and uric acid content in Chinese chicken broth can provide consumers with scientific dietary information, especially those with gout.

Currently, a variety of methods have been established for the determination of purines in foods, including ion chromatography [15], capillary electrophoresis [16], enzyme-assisted electrochemical detection [17], and high-performance liquid chromatography (HPLC) [18,19]. Among these methods, HPLC is the mainstream detection method. It has been widely used to determine purine content in animal and plant foods, fungi and algae, and aquatic products due to its advantages of simplicity, efficiency, and accuracy [2,20]. Despite chicken broth being one of the most consumed broths worldwide, to our knowledge, the content of purine in chicken soup products has not been determined in any of the previous studies. The reason may be that the variety of nutrients such as proteins, amino acids, and fats in chicken broth makes the pretreatment process complex and challenging to remove interfering substances altogether, significantly impacting the accurate quantification of purines. It is also worth noting that there is a lack of attention to uric acid levels in food. Few studies have reported on uric acid levels in food, which seriously hinders the overall assessment of the palatability of food for the gout population. In this study, an optimal trifluoroacetic acid/formic acid (TFA/FA) hydrolysis condition and reliable HPLC conditions were established to simultaneously determine four purines and uric acid in Chinese chicken soup. The present study aimed to fill the gap in detecting purines and uric acid in Chinese chicken broth and strengthen the meal management for patients with hyperuricemia and gout.

## 2. Materials and Methods

### 2.1. Chemicals and Reagents

Purine standards (adenine, guanine, hypoxanthine, and xanthine, purity ≥ 99%) were obtained from Sigma-Aldrich Co., Ltd. (St. Louis, MO, USA). Uric acid standard (purity ≥ 98%) was provided by Shanghai Yuanye Biotechnology Co., Ltd. (Shanghai, China). HPLC-grade TFA and FA were purchased from Shanghai Aladdin Biochemical Technology Co., Ltd. (Shanghai, China). HPLC-grade methanol was supplied by Merck (Darmstadt, Germany). Guaranteed reagent grade perchloric acid (PCA) was provided by Yonghua Chemical Co., Ltd. (Jiangsu, China). Guaranteed reagent grade for potassium dihydrogen phosphate (KH_2_PO_4_), phosphoric acid (H_3_PO_4_), and analytical grade sodium hydroxide (NaOH), and potassium hydroxide (KOH) were all acquired by Sinopharm Chemical Reagent Co., Ltd. (Shanghai, China). Ultrapure water was supplied by Sartorius-Arium pro system (Sartorius AG, Goettingen, Germany).

### 2.2. Sample Collection

Yellow-feather chickens 400 days old (gutted) were supplied by the Wens Foodstuff Group Co., Ltd. (Guangdong, China) and stored at −20 °C. A total of 12 commercially available Chinese chicken broths (BR-1, BR-2, BR-3, BR-4, BR-5, BR-6, BR-7, BR-8, BR-9, BR-10, BR-11, and BR-12) were purchased from different provinces in China, including BR-1, BR-4, BR-5, BR-8, and BR-10 from Hubei; BR-2 and BR-11 from Guangdong; BR-3 from Guizhou; BR-6 from Jiangsu; BR-7 from Fujian; BR-9 from Yunnan; and BR-12 from Jiangxi. The major ingredients of chicken broth products included old hen, salt, and water.

### 2.3. Standard Solutions Preparation

Twenty-five mg of each analyte of adenine, guanine, hypoxanthine, xanthine, and uric acid standards was weighed and transferred into a 50-mL volumetric flask containing 40 mL ultrapure water, and 5 mL of 0.1 M NaOH solution was added to aid dissolution. Then, ultrapure water was used to dilute the samples to configure as an individual standard stock solution at the concentration of 500 mg/L and stored at 4 °C for one week.

The combined standard stock solution at the concentration of 100 mg/L for each analyte was prepared by mixing equal volumes (5 mL) of an individual standard stock solution of adenine, guanine, hypoxanthine, xanthine, and uric acid with the concentration of 500 mg/L and stored at 4 °C until use. The standard working solution used in the study was freshly prepared by diluting the combined standard stock solution to the desired concentration with ultrapure water.

### 2.4. Liquid Chromatographic Conditions

The separation and quantification of the four purines and uric acid were performed using an Agilent 1260II ultra-HPLC (Agilent Technologies, Santa Clara, CA, USA) equipped with a G7104C quaternary pump, G7129C autosampler, G7116A column heater, and variable wavelength detector (VWD). An Agilent TC-C18 (2) (4.6 mm × 250 mm, 5.0 µm) was used as the analytical column, and the injection volume was 10 µL. In order to establish a suitable HPLC detection method, the following parameters: mobile phase (7 × 10^−3^ M KH_2_PO_4_, 0.02 M KH_2_PO_4_, and methanol-water solutions); flow rate (0.8, 1.0 and 1.2 mL/min); column temperature (25 °C, 28 °C, 30 °C, and 35 °C) and absorption wavelength have been optimized. Data were collected and processed using OpenLab CDS Data Analysis software (product version 2.5; Agilent Technologies, Santa Clara, CA, USA).

### 2.5. Chinese Chicken Broth Preparation

Chinese chicken stock was prepared according to the protocol of Qi et al. [21]. Briefly, yellow-feather chicken carcasses with the head, claws, and internal organs removed were cut evenly in half along the back, rinsed and drained in cold water, and cooked in a 5.6 L stainless steel stockpot (RT22AA1, Supor Co., Ltd., Yuhuan, China) with purified water (Yibao, Shenzhen, China) at the meat to water ratio of 1:2 (*w*/*w*). The chicken carcass was placed in the stainless steel pot when the purified water was at 95–99 °C, and cooking was timed when the broth temperature again reached 95–99 °C. After 4 h of stewing, the top layer of fat was removed and the chicken stock was stored at −20 °C for further analysis.

### 2.6. TFA/FA Hydrolysis

In order to improve the efficiency, yield, and recovery of purine extraction from chicken broth, this study was conducted to optimize the conditions for the extraction of purine and uric acid from Chinese chicken stock through a single-factor test combined with an orthogonal experimental. The original extraction method for purines and uric acid in chicken broth was referenced as follows. The 1 mL of chicken broth was taken in a centrifuge tube containing 10 mL of TFA/FA blended acid (1:1, *v/v*). Samples were heated by shaking in a water bath at 90 °C for 12 min and then cooled rapidly in an ice bath. Subsequently, the hydrolysate in the centrifuge tube was blown to near dryness with nitrogen. The residue was reconstituted with 4 mL of 0.02 M KH_2_PO_4_ (pH 4.0) solution. The mixture was vortexed for 1 min (Vortex Genius 3, IKA-Werke GmbH & Co., Staufen, Germany) and dissolved using ultrasound assistance (XO-25-12DTD, Nanjing Xianou Instruments Manufacture Co., Ltd., Nanjing, China). Finally, the solution was filtered through a hydrophilic PTFE-Q type (13 mm × 0.22 µm) filter membrane (Agilent Technologies, Santa Clara, CA, USA) and injected into the HPLC system analysis.

#### 2.6.1. Single-Factor Test

Hydrolysis was conducted as described above, and the effect of several independent factors on purine and uric acid extraction from the chicken broth was investigated by a single-factor test, which included the following parameters: hydrolysis temperature (60 °C, 65 °C, 70 °C, 75 °C, 80 °C, 85 °C, 90 °C, and 95 °C); hydrolysis time (15, 20, 25, 30, 35, 40, 45 and 50 min); TFA and FA concentration (10%, 20%, 30%, 40%, 50%, 60%, 70% and 80%); chicken broth-TFA/FA (1:1, *v/v*) volume ratio (1:1, 1:3, 1:5, 1:7, 1:10, 1:12, 1:15, and 1:20). When one factor is studied, the other factors should remain constant. The basis index of the single-factor test was the total extraction rate, which refers to the sum of the extraction rate of each analyte of adenine, guanine, hypoxanthine, xanthine, and uric acid. The extraction rate was calculated as follows:

(1) $$\mathrm{Extraction}\;\mathrm{rate}\;\left(\%\right)=\frac{\mathrm{CV}\times 10^{-3}}{M}\times 100\%$$

where C (mg/L) is the concentration of each analyte of adenine, guanine, hypoxanthine, xanthine, and uric acid; V (L) is the volume of the dissolved residue, which is 4 mL; M (g) represents the mass of 1 mL chicken broth sample.

#### 2.6.2. Orthogonal Experimental Design

Analysis of variance (ANOVA) was performed using SAS software (SAS Inst. Inc., Cary, NC, USA) on the results of the single-factor test. The statistical difference with a 99% confidence limit at *p* < 0.01 was used. The factors that significantly impacted the extraction of purine and uric acid were selected based on the analysis results and further optimized by orthogonal experimental. The orthogonal experiment design is shown in Table 1, and each factor in the orthogonal experimental design was studied at three different levels near the maximum response value.

### 2.7. PCA Hydrolysis

The 1 mL of chicken broth was taken in a centrifuge, and then 3 mL of 10% PCA was added and mixed via vortexing for 30 s. The mixture was hydrolyzed in a boiling water bath for 60 min. Then, the extract was chilled in ice, and the pH of which was adjusted to 7.0 with a potassium hydroxide solution with the concentration of 2.0 mol/L and 5.0% phosphoric acid. Finally, the pH of the extract was adjusted to 4.0 with 5.0% phosphoric acid and then diluted to 8 mL with 0.02 M KH_2_PO4 (pH 4.0) solution, mixed for 30 s, and centrifuged at 2000 r/s for 2 min. Finally, the solution passed through a 0.22 µm hydrophilic filter membrane (Agilent Technologies, Santa Clara, CA, USA) and then injected into the HPLC system for analysis.

### 2.8. Method Validation

#### 2.8.1. Determination of Linearity, Limit of Detection, and Limit of Quantification

Each standard was determined between 0.05 and 100 mg/L using seven calibration levels (0.05, 0.2, 1.0, 5, 25, 50, and 100 mg/L) in six times. These concentration points were obtained by diluting the stock solution of combined standards with the concentration of 100 mg/L for each analyte with ultrapure water. The linearity of response was performed using plotting peak areas against the concentration of the standard and evaluated by the squared correlation coefficient (R^2^). The R^2^ close to 1.0 indicates smaller dispersion of the experimental points and more excellent reliability of the estimated regression coefficients [22]. For the calculation of the limit of detection (LOD), the signal-to-noise ratio (S/N) was monitored until an S/N ratio of 3:1 was reached. The limit of quantification (LOQ) was estimated at an S/N of 10 [23].

#### 2.8.2. Verification of Precision, Repeatability, and Accuracy

The precision of the method and the repeatability of the purine and uric acid extraction process were evaluated by testing the mixed standard solution and the extraction sample from Chinese chicken broth six times, and the relative standard deviation (RSD) was calculated. Sample spiked recovery experiments were performed to assess the accuracy of the method. Accuracy of the method was determined by the addition of known amounts of purine and uric acid standards (*n* = 3, at each level of 50%, 100%, and 200% levels) into Chinese chicken broth samples, which was then processed under optimal pretreatment conditions and then analyzed according to the chromatographic conditions determined in Section 3.1. In this work, the mean recoveries of 100 ± 10% for adenine, guanine, xanthine, and uric acid and 100 ± 5% for hypoxanthine were acceptable.

## 3. Results and Discussion

### 3.1. Optimization of HPLC Conditions

To establish an appropriate HPLC detection method, several conditions such as mobile phase, column temperature, flow rate, and detection wavelength were assayed in this study.

Different mobile phases such as 7 × 10^−3^ M KH_2_PO_4_, 0.02 M KH_2_PO_4_, and methanol-water (2:98, *v/v*) solutions were tried. Initially, regardless of column temperature, flow rate, and other related parameters, only the 0.02 M KH_2_PO_4_ solution separated the peaks of the four purines and uric acid. Furthermore, the best resolution was achieved when the pH of the 0.02 M KH_2_PO_4_ solution was adjusted to 4.0 with H_3_PO_4_. Therefore, 0.02 M KH_2_PO_4_ (pH 4.0) was determined as the mobile phase. The Agilent TC-C18 (2) column was used for the chromatographic analysis as it has excellent separation properties for polar compounds, basic compounds, and outstanding aqueous mobile phase stability.

Regarding optimizing the column temperature, the analysis of different temperatures (25 °C, 28 °C, 30 °C, and 35 °C) showed that the five compounds were better separated and had the highest peak area response when the temperature was set at 30 °C. Additionally, the assay of different flow rates (0.8, 1.0, and 1.2 mL/min) showed that a flow rate of 1.0 mL/min effectively reduced the analysis time and improved the separation of the compounds.

**Figure 2:**
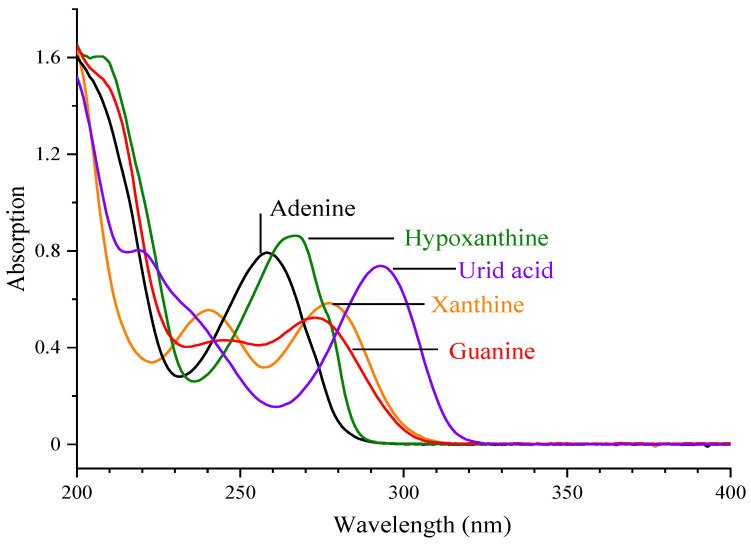
UV full wavelength scan of purine bases and uric acid.

The UV full spectrum scans of the five compounds are shown in Figure 2. Adenine, guanine, hypoxanthine, xanthine, and uric acid have characteristic absorption peaks at 258, 245, 267, 240, and 293 nm, respectively. Based on previous reports on the detection of purine and uric acid, the most commonly used UV absorption wavelengths were 254 [24,25] and 260 nm [2,26]. In this study, detection wavelengths of 254 and 260 nm were tested, respectively. The results highlight the high peak area response, better separation, and shorter analysis time of the five compounds at a wavelength of 254 nm. Therefore, 254 nm was chosen as the detection wavelength for the final experiment.

**Figure 3:**
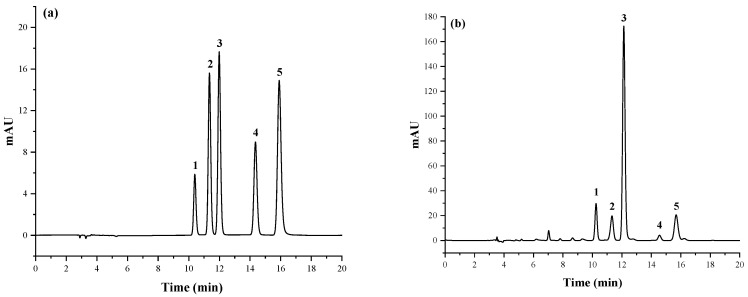
High-performance liquid chromatography (HPLC) profiles of four purine bases and uric acid at 254 nm. (**a**) Mixed standard working solution (5 mg/L); (**b**) Chinese chicken soup sample. Column: Agilent TC-C18 (2) (4.6 mm × 250 mm, 5.0 µm). Mobile phase: 0.2 M KH_2_PO_4_ buffer solution, pH = 4. Flow rate: 1.0 mL/min. Column temperature: 30 °C, injection column: 10 µL. Peaks: 1, Uric acid; 2, Guanine; 3, Hypoxanthine; 4, Xanthine; 5, Adenine.

Based on the above optimized chromatographic conditions, an HPLC method for the simultaneous determination of purine and uric acid in Chinese chicken broth was developed. The chromatograms of the combined standard solutions and the chicken broth samples are shown in Figure 3. The five compounds showed short peak times, good separation, and no excessive impurity interference around the retention time of each target compound in the chicken broth samples.

### 3.2. Optimization of Sample Pretreatment

#### 3.2.1. Single-Factor Test

An ideal acid hydrolysis method should completely hydrolyze bound purines such as nucleic acids, nucleotides, and nucleosides in foods to free purine bases without further degradation. Among the available reports, PCA and TFA/FA are the most typically used acid hydrolysis reagents [27]. Using Chinese chicken broth as the subject of our study, we compared the hydrolysis of the two acids by pre-experiments. The results showed that the PCA method had a longer hydrolysis time and lower total extraction rate than the TFA/FA method. The neutralization reaction between KOH and PCA hydrolysate also produced a precipitate after the hydrolysis was completed. In addition, some researchers have confirmed that high concentrations of PCA can induce the degradation of purine bases [28]. All of the above factors can affect the accurate quantification of purine bases. In the present study, TFA/FA with high hydrolysis capacity and the low impact was chosen as the acid hydrolysis reagent, and the hydrolysis conditions were further optimized by a single-factor test combined with an orthogonal design.

**Figure 4:**
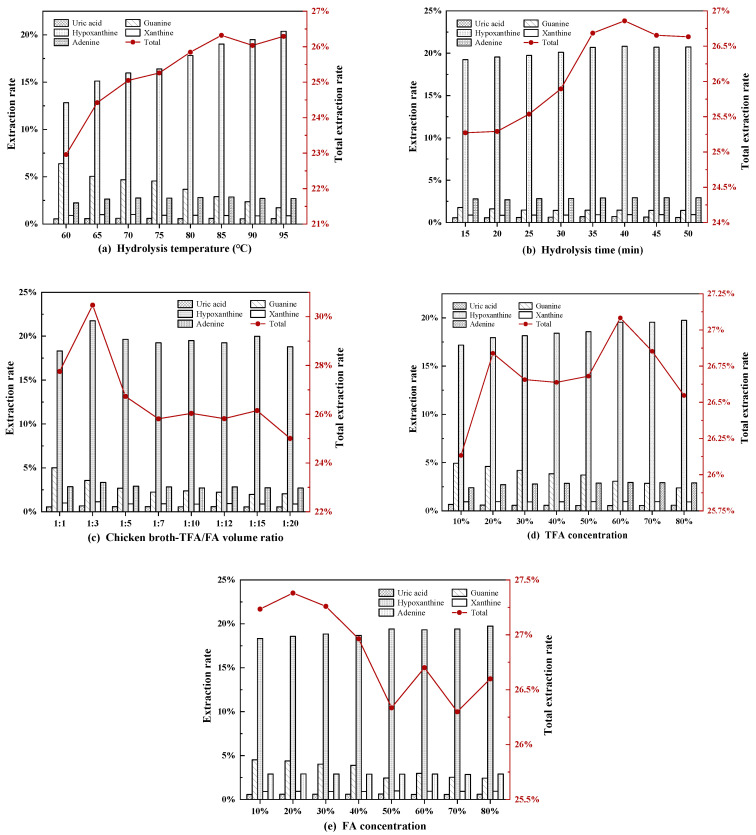
Effect of different factors on extraction rate using the trifluoroacetic acid/formic acid (TFA/FA) method. (**a**) Hydrolysis temperature; (**b**) Hydrolysis time; (**c**) Chicken broth-TFA/FA (1:1, *v/v*) volume ratio; (**d**) TFA concentration; (**e**) FA concentration. The left vertical axis corresponds to the extraction rate of each analyte of adenine, guanine, hypoxanthine, xanthine and uric acid, and the right vertical axis corresponds to the total extraction rate of the five analytes.

Many parameters can influence the purines and uric acid extraction from Chinese chicken broth, such as the time and temperature of hydrolysis, the chicken broth-TFA/FA (1:1, *v/v*) volume ratio, and the TFA and FA solution concentrations. Those parameters were studied in this work for extraction process optimization of Chinese chicken broth (Figure 4). According to the results presented in Figure 4a, the total extraction rate of purine and uric acid gradually increased with hydrolysis temperature. The higher total extraction rate was obtained for the temperature reaching 85 °C, beyond which the total extraction rate decreased slightly. This is because some of the purine bases will be destroyed under high-temperature conditions [29]. According to the results presented in Figure 4b, the increase in the initial hydrolysis time increased the total extraction rates. At the hydrolysis time of 40 min, the total extraction rate has a maximum value. For less time than this, the bound purines are not entirely. The chicken broth-TFA/FA (1:1, *v/v*) volume ratio refers to the ratio of the volume of chicken broth to the sum of the volume of TFA and FA. The total extraction rate of purine and uric acid fluctuated with the chicken broth-TFA/FA (1:1, *v/v*) volume ratio, with a maximum total extraction rate of about 30.5%. In addition, Figure 4d,e shows that the TFA/FA method had a better hydrolysis effect when the TFA concentration was 60% and the FA concentration was 20%.

#### 3.2.2. Orthogonal Experiment Design

ANOVA was performed on the results of the single-factor test. The analysis indicated that hydrolysis temperature, hydrolysis time, and FA concentration had a significant influence on purine and uric acid extraction (*p* < 0.01), which was selected for the three-factor and three-level orthogonal optimization experiment. The remaining factors were maintained at the optimum level of the single-factor test, meaning the chicken broth-TFA/FA (1:1, *v/v*) volume ratio of 1:3 and the TFA concentration of 60%.

The orthogonal experiment results are shown in Table 2, and the K and R values were calculated. K refers to the average of the results of each column of factors at the same level, and R is the range of the average of the results of each column of factors at the same level. The larger the R-value, the greater the influence of this factor on the test index. Orthogonal experiment results showed that the factors affecting the total extraction rate of purine and uric acid from chicken broth were ranked as A (hydrolysis temperature) > B (hydrolysis time) > C (FA concentration). The best combination obtained by statistical analysis of the experimental results was A (85 °C) B (40 min) C (20%), which is inconsistent with the optimal combination in the actual treatment (Experimental No.5, A (85 °C) B (40 min) C (30%)). The verification test was carried out with the combination of A (85 °C) B (40 min) C (20%) and A (85 °C) B (40 min) C (30%). The result was A (85 °C) B (40 min) C (20%): 27.33 mg/100 g > A (85 °C) B (40 min) C (30%): 27.31 mg/100 g. Therefore, the final optimum hydrolysis conditions for the TFA/FA method were determined: hydrolysis temperature 85 °C, hydrolysis time 40 min, the concentration of TFA is 60%, the concentration of FA is 20%, and chicken broth-TFA/FA (1:1, *v/v*) volume ratio of 1:3.

### 3.3. Validation of Analytical Methods

#### 3.3.1. Linearity of the Standard Curves, the Limit of Detection, and the Limit of Quantification

The linearity, linear ranges, LODs, and LOQs of five compounds were performed using the developed HPLC method (Table 3). The squared correlation coefficients (R^2^ > 0.9997) indicated excellent linearity between the concentrations of adenine, guanine, hypoxanthine, xanthine, and uric acid and their peak areas in the range of 0.05 to 100 mg/L. The LODs ranged from 0.58 to 1.71 µg/L, and the LOQs were less than 5.70 µg/L. The method has a lower LOD and LOQ than previously reported to determine purine and uric acid [30,31,32].

#### 3.3.2. Precision, Repeatability, and Accuracy

Mixed standards were used to assess the precision of the method. Chinese chicken broth samples were prepared using the optimized pretreatment method described above for reproducibility and recovery tests. As shown in Table 3, the RSDs for all five compounds were 1%, indicating that the instrumental assay established in this study was of good precision. The repeatability of the method RSD ranged from 1% to 2%, all with good reproducibility. Recovery of the method was performed by spiking known amounts of purine and uric acid standards to Chinese chicken stock and then analyzing both spiked and nonspiked samples in triplicate. The average recoveries of uric acid, guanine, hypoxanthine, xanthine, and adenine were 93%, 101%, 99%, 91%, and 95%, respectively, and the RSDs were all less than 3% (Table 4). The recovery rate of this experiment is consistent with the result that M Piñeiro-Sotelo et al. [33] used the TFA/FA method (1:1, *v/v*) to hydrolyze samples with a recovery rate greater than 90%. The experiments showed that the optimized TFA/FA method for hydrolysis of chicken broth samples was less destructive to purine bases. The purine and uric acid contents were determined with high accuracy and reproducibility.

### 3.4. Application to Commercially Available Chinese Chicken Broths

The optimum chicken broth pretreatment conditions and the HPLC method developed above were applied to determine the purine and uric acid contents of the 12 most popular Chinese chicken broths currently available on the market (Table 5). Total content refers to the sum of the four purines and uric acid content. The total purine and uric acid contents in all chicken soup samples ranged from 94.04 to 363.26 mg/L. The highest total content was observed in chicken stock BR-1 (363.26 ± 4.65 mg/L) from Wuhan, Hubei Province, followed by chicken stock BR-2 (322.47 ± 3.38 mg/L). The least total content was detected in chicken soups BR-10 (120.53 ± 2.11 mg/L) and BR-12 (94.04 ± 1.49 mg/L). The total purine content in the 12 Chinese chicken broths ranged from 93.64 to 359.40 mg/L. Compared to previous studies, the purine content of chicken soup was remarkably higher than that of high purine beverages such as beer and soybean milk [18,34]. In general, the total purine and uric acid content and total purine content of different commercially available chicken soups were mostly significantly different (*p* < 0.05). The different results could be due to the different types, ages and processing methods of raw chickens. According to the American College of Rheumatology guideline for the management of gout, selective restriction of purine intake is recommended for patients with gout, regardless of disease activity [35]. In Japan, the guideline for gout and hyperuricemia limits purine intake to 400 mg/d [36]. Therefore, patients with gout and hyperuricemia should avoid chicken broth with high purine content in BR-1 and BR-2. The novel HPLC method is reliable for application according to commercial sample analysis.

Table 5 shows that there was a significant difference in the levels of the four purines in the various Chinese chicken broths (*p* < 0.05). The highest content in the chicken broth was hypoxanthine, which accounted for 54.92% of the total purine content. Guanine and adenine were similar in scope, and xanthine was the least abundant. The proportions of the four purines are consistent with previous animal food test results [37]. It is noteworthy that all 12 commercial chicken soups contained uric acid, which is present at low levels but can directly affect the body’s blood uric acid levels when ingested with the chicken soup. Theoretically, the ability of each purine to convert into uric acid is equivalent. However, the Clifford oral purine experiment showed that hypoxanthine and adenine had a stronger ability to raise serum uric acid, while guanine and xanthine had no significant effect [38]. In conclusion, given the uric acid and high purine content of chicken broth and the dominant blood uric acid effect of hypoxanthine, the chicken broth should be avoided in patients in the acute phase of gout even if it is nutritious.

## 4. Conclusions

Gout is the second most common metabolic disease affecting human health after diabetes. Gout is closely related to the content of exogenous purines and uric acid consumed in the diet. Chinese chicken broth is a delicious traditional meat soup, rich in protein, fat, minerals, and other nutrients, and is popular among consumers. Chicken broth is often considered a high purine food, posing a health risk to patients with gout and hyperuricemia. This work copes with the optimization of purines and uric acid content simultaneous determination in Chinese chicken stock when hydrolysis and extraction procedures, as well as HPLC, were studied. The peak time of the five analytes was within 20 min, the LODs varied between 0.58 and 1.71 µg/L, the LOQ between 1.92 and 5.70 µg/L, the precision RSDs were 1%, and the spiked recoveries were 91–101%. The complete assay had good precision, accuracy, and sensitivity. It has been demonstrated that Chinese chicken broth contains a significant amount of purines, with the highest content being hypoxanthine. It is also worth noting that some uric acid is also present in chicken soup, which can directly affect the body’s blood uric acid levels when ingested.

## Figures and Tables

**Table 1:** Chicken broth pretreatment orthogonal test.

Level	Factor		
	Hydrolysis Temperature (°C)	Hydrolysis Time (min)	FA Concentration (%)
1	80	35	10
2	85	40	20
3	90	45	30

**Table 2:** Results of the chicken broth pretreatment orthogonal test (*n* = 3).

Experimental No.	A (°C)	B (min)	C (%)	Total Content (mg/100 g)
1	1(80)	1(35)	1(10)	26.90
2	1(80)	2(40)	2(20)	26.99
3	1(80)	3(45)	3(30)	27.13
4	2(85)	1(35)	2(20)	27.14
5	2(85)	2(40)	3(30)	27.31
6	2(85)	3(45)	1(10)	26.68
7	3(90)	1(35)	3(30)	26.07
8	3(90)	2(40)	1(10)	26.64
9	3(90)	3(45)	2(20)	26.63
K1	27.00	26.70	26.74	
K2	27.04	26.98	26.92	
K3	26.47	26.81	26.84	
R	0.60	0.28	0.18	
Factor priorities	A > B > C			
Optimal combination	A2B2C2: A (85 °C) B (40 min) C (20%)			

A: hydrolysis temperature; B: hydrolysis time; C: FA concentration; Total content: the mean of total purine and uric acid contents; K: the average of the results of each column of factors at the same level; R: the range of the average of the results of each column of factors at the same level.

**Table 3:** Calibration curves, limits of detection (LODs), limits of quantification (LOQs), precision and repeatability for the four purines and uric acid using the optimized HPLC method (*n* = 6).

Compound	Regression Equation	R^2^	Linear Range (mg/L)	LOD (µg/L)	LOQ (µg/L)	Precision (%RSD)	Repeatability (%RSD)
Uric acid	y = 12.2198x + 2.0876	0.9998	0.05–100	1.71	5.70	1	1
Guanine	y = 36.9705x − 1.0485	0.9998	0.05–100	0.64	2.13	1	1
Hypoxanthine	y = 40.1194x + 11.6305	0.9997	0.05–100	0.58	1.92	1	1
Xanthine	y = 25.0866x + 5.5252	0.9998	0.05–100	1.14	3.79	1	2
Adenine	y = 50.9974x + 10.7899	0.9997	0.05–100	0.66	2.19	1	1

R^2^: squared correlation coefficient; RSD: relative standard deviation.

**Table 4:** Recoveries of four purines and uric acid (*n* = 3).

Compound	Original Quantity (mg)	Spiked Quantity (mg)	Measured Quantity (mg)	Recovery (%)	RSD (%)	Average Recovery (%)
Uric acid	0.0058	0.0029	0.0084	92	1	93
		0.0058	0.0111	92	1	
		0.0115	0.0167	95	2	
Guanine	0.0318	0.0159	0.0484	104	2	101
		0.0318	0.0627	97	3	
		0.0636	0.0963	101	3	
Hypoxanthine	0.1959	0.0980	0.2922	98	2	99
		0.1959	0.3892	99	1	
		0.3919	0.5861	100	1	
Xanthine	0.0098	0.0049	0.0143	92	2	91
		0.0098	0.0188	91	2	
		0.0196	0.0278	92	2	
Adenine	0.0295	0.0147	0.0434	95	2	95
		0.0295	0.0571	94	2	
		0.0580	0.0854	97	3	

**Table 5:** Quantitative results of the determination of purines and uric acid in commercial Chinese chicken broths (*n* = 3).

Sample	Average Concentration ± SD						
	Uric Acid (mg/L)	Guanine (mg/L)	Hypoxanthine (mg/L)	Xanthine (mg/L)	Adenine (mg/L)	Total Purine (mg/L)	Total Amount (mg/L)
BR-1	3.86 ± 0.04 ^Fc^	71.92 ± 1.14 ^Cb^	223.33 ± 2.84 ^Ba^	9.19 ± 0.10 ^Eb^	54.97 ± 0.80 ^Da^	359.40 ± 4.65 ^Aa^	363.26 ± 4.65 ^Aa^
BR-2	4.42 ± 0.46 ^Gb^	85.95 ± 0.79 ^Da^	180.84 ± 1.91 ^Cb^	12.87 ± 0.54 ^Fa^	38.40 ± 0.25 ^Ec^	318.05 ± 3.43 ^Bb^	322.47 ± 3.38 ^Ab^
BR-3	3.80 ± 0.01 ^Dc^	60.75 ± 1.40 ^Cd^	144.12 ± 2.46 ^Bc^	3.96 ± 0.33 ^De^	55.49 ± 1.48 ^Ca^	264.31 ± 5.60 ^Ac^	268.12 ± 5.59 ^Ac^
BR-4	1.80 ± 0.07 ^Ed^	43.53 ± 1.08 ^Cf^	144.75 ± 3.69 ^Bc^	5.46 ± 0.34 ^Ed^	33.60 ± 0.73 ^Dd^	227.35 ± 5.84 ^Ad^	229.15 ± 5.83 ^Ad^
BR-5	1.65 ± 0.14 ^Ed^	66.47 ± 0.73 ^Cc^	112.54 ± 1.13 ^Be^	3.57 ± 0.45 ^Ee^	41.37 ± 0.39 ^Db^	223.95 ± 2.59 ^Ad^	225.60 ± 2.46 ^Ad^
BR-6	0.74 ± 0.03 ^De^	43.10 ± 1.02 ^Cf^	138.14 ± 2.22 ^Bd^	1.18 ± 0.24 ^Df^	39.49 ± 0.65 ^Cc^	221.92 ± 4.10 ^Ad^	222.66 ± 4.08 ^Ad^
BR-7	1.78 ± 0.11 ^Dd^	34.91 ± 1.55 ^Cg^	97.99 ± 4.59 ^Bf^	5.25 ± 0.24 ^Dd^	34.86 ± 1.52 ^Cd^	173.01 ± 7.90 ^Ae^	174.79 ± 8.01 ^Ae^
BR-8	0.81 ± 0.03 ^De^	32.72 ± 0.71 ^Ch^	92.22 ± 2.20 ^Bg^	1.68 ± 0.12 ^Df^	29.67 ± 0.72 ^Ce^	156.28 ± 3.64 ^Af^	157.09 ± 3.65 ^Af^
BR-9	0.87 ± 0.04 ^Ee^	31.88 ± 0.51 ^Ch^	77.42 ± 1.32 ^Bh^	1.48 ± 0.05 ^Ef^	27.77 ± 0.78 ^Df^	138.55 ± 2.46 ^Ag^	139.42 ± 2.44 ^Ag^
BR-10	1.66 ± 0.06 ^Ed^	50.16 ± 1.14 ^Be^	44.77 ± 0.45 ^Cj^	1.23 ± 0.06 ^Ef^	22.71 ± 0.57 ^Dg^	118.87 ± 2.17 ^Ah^	120.53 ± 2.11 ^Ah^
BR-11	6.84 ± 0.28 ^Fa^	22.90 ± 0.32 ^Di^	70.03 ± 0.39 ^Ci^	6.77 ± 0.25 ^Fc^	16.59 ± 0.28 ^Eh^	116.29 ± 0.55 ^Bh^	123.13 ± 0.82 ^Ah^
BR-12	0.39 ± 0.06 ^Ef^	32.67 ± 0.17 ^Ch^	37.38 ± 0.05 ^Bk^	1.34 ± 0.06 ^Ef^	22.25 ± 1.31 ^Dg^	93.64 ± 1.43 ^Ai^	94.04 ± 1.49 ^Ai^

SD: standard deviation. Different capital letters in the same row indicate significant differences between the contents of different compounds in the same Chinese chicken broth (*p* < 0.05), and different lowercase letters in the same column indicate significant differences between different samples of the same compound (*p* < 0.05).

## Data Availability

Data are contained within the article.
